# CDC5L facilitates cardiomyocyte proliferation and ameliorates myocardial ischemia-reperfusion injury via modulation of the FGF10-YAP axis: CDC5L facilitates cardiomyocyte proliferation and ameliorates myocardial I/R injury

**DOI:** 10.3724/abbs.2025213

**Published:** 2025-11-14

**Authors:** Tianwen Wei, Tangjiang Wan, Yuxiao Sun, Yucheng Liang, Zhihao Lin, Shitong Shen, Qi Zhang, Mengli Chen, Yafei Li

**Affiliations:** 1 Department of Cardiology Shanghai East Hospital School of Medicine Tongji University Shanghai 200120 China; 2 Department of Cardiology the Affiliated Suzhou Hospital of Nanjing Medical University Suzhou Municipal Hospital Gusu School Nanjing Medical University Suzhou 215000 China

**Keywords:** cardiac ischemia-reperfusion, cardiomyocyte proliferation, apoptosis, CDC5L, FGF10, YAP

## Abstract

Myocardial infarction (MI) causes irreversible cardiomyocyte loss, creating a need for cardiac repair therapies. The role of cell division cycle 5-like (CDC5L), a cell cycle regulator, in cardiac repair is unknown. This study aims to define the role of CDC5L in mitigating ischemia-reperfusion (I/R) injury by assessing its impact on cardiomyocyte proliferation and apoptosis and to determine the mechanism involving the FGF10-YAP axis. We model cardiac injury using *in vitro* oxygen-glucose deprivation/reoxygenation (OGD/R) in neonatal mouse cardiomyocytes and *in vivo* I/R in adult mice. To investigate CDC5L function, we modulate its expression via adenoviral or AAV9-mediated overexpression or knockdown. Proliferation markers (EdU ^+^, Ki67 ^+^, pH3 ^+^), apoptosis (TUNEL staining, Bax/Bcl-2 ratio), and cardiac function (echocardiography) are assessed. Through transcriptomic screening, we identify CDC5L downstream targets and validate their functional roles using *FGF10* knockdown rescue assays. We find that CDC5L is upregulated in the post-I/R murine myocardium. Its overexpression enhances cardiomyocyte proliferation, preserves cardiac function, reduces apoptosis, and diminishes infarct size. Transcriptomic analysis identifies FGF10 as a key downstream effector, and we confirm that CDC5L upregulates FGF10 expression. Notably, *FGF10* knockdown reverses the proliferative and anti-apoptotic effects of CDC5L. Moreover, the CDC5L-mediated reduction in YAP phosphorylation is also dependent on FGF10, as this effect is abolished upon *FGF10* knockdown. In conclusion, CDC5L attenuates cardiac I/R injury by promoting cardiomyocyte proliferation and inhibiting apoptosis through the FGF10-YAP pathway. This CDC5L-FGF10-YAP axis represents a promising therapeutic target to improve myocardial regeneration and recovery after myocardial infarction.

## Introduction

Myocardial infarction (MI), a serious cardiovascular disease caused by sudden interruption of coronary blood flow, directly results in the loss of cardiomyocytes and triggers complex pathological processes, including myocardial remodelling and heart failure, ultimately leading to death [ 1, 2]. At present, the treatment for myocardial infarction focuses mainly on ischemia-reperfusion (I/R) methods, such as percutaneous coronary intervention, coronary artery bypass grafting, and drug thrombolysis [3]. Although these methods improve the blood supply of ischemic myocardial tissue after MI, they do not rescue necrotic myocardial cells or regenerate reduced cardiomyocytes [4]. As the ultimate treatment, heart transplantation completely resolves severe heart failure caused by heart injury, but the lack of donors limits its clinical application [5]. Therefore, elucidating the endogenous mechanisms that reactivate cardiomyocyte proliferation and mitigate apoptosis represents a promising therapeutic avenue for improving cardiac repair after MI.

A key characteristic of adult mammalian cardiomyocytes is their exit from the cell cycle and loss of proliferative function. Therefore, when the myocardium is subjected to ischemic injury, necrotic myocardial cells can not be replaced and repaired by newly formed normal myocardial cells, and the tissue in the infarcted area is replaced only by fibrous scars [6]. This fibrous scar tissue does not have normal myocardial function, resulting in severe damage to cardiac function. Numerous studies have shown that the hearts of newborn mice regenerate within 7 days after birth, but this regenerative ability is lost after 7 days, indicating that there is a brief window for effective regeneration of myocardial cells [7]. Scholars have extensively explored and overcome the limitations of the endogenous regeneration of myocardial cells outside the regenerative window from multiple perspectives. (1) Overexpression of the OSKM transcription factor in the adult myocardium for a short period of time induces cardiomyocyte dedifferentiation and reprogramming, allowing cardiomyocytes to re-enter the cell cycle, promote cardiac regeneration and promote repair after myocardial infarction [8]. (2) HMGCS2 overexpression increases the dedifferentiation and proliferation of adult myocardial cells after myocardial infarction to improve cardiac function, and *HMGCS2* knockdown worsens cardiac function after myocardial infarction reperfusion. (3) Inhibition of succinate dehydrogenase (SDH) activity prolongs the regeneration window of neonatal mouse hearts and promotes myocardial regeneration and repair after myocardial infarction [ 9 , 10]. These findings indicate that expanding the myocardial regeneration window and exploring new methods to trigger cell cycle re-entry in adult cardiomyocytes may provide new perspectives for the treatment of adult myocardial infarction.

Cell division cycle 5-like (CDC5L) is a DNA-binding protein involved in cell cycle control, and it acts as a transcriptional activator and plays a role in mRNA precursor splicing [11]. In addition, as a core component of the PRP19-CDC5 complex, CDC5L also participates in the repair response after DNA damage [12]. Previous studies have shown that CDC5L promotes cancer cell proliferation, inhibits cancer cell apoptosis, and promotes cancer progression. For example, CDC5L overexpression induces the phosphorylation of ERK1/2 and JAK2, increases the proportion of cells in the G2/M phase, promotes the proliferation of non-small cell lung cancer cells, and inhibits tumor cell apoptosis [13]. In contrast, knocking down *CDC5L* inhibits proliferation and induces apoptosis in gliomas and bladder cancer [14]. A previous study revealed that CDC5L is important for maintaining the proliferation ability of myoblasts and that it promotes chondrocyte regeneration by regulating the pre-mRNA splicing of nuclear transcription factors [15]. However, the potential role of CDC5L in cardiac repair post-MI remains unexplored. Given its established functions in cell cycle regulation and apoptosis inhibition in other cell types, we hypothesized that CDC5L promotes cardiomyocyte proliferation and survival following I/R injury. In this study, this hypothesis was investigated to identify the signaling pathway involved, aiming to define CDC5L as a novel therapeutic target for enhancing myocardial regeneration. We constructed *in vitro* and *in vivo* models using OGD/R-induced cardiomyocytes and I/R-treated adult mice. Moreover, we detected the expression of CDC5L and explored the role of CDC5L in cell proliferation and apoptosis after OGD/R and MI. Finally, we investigated the potential underlying molecular mechanisms to provide a new perspective for the prevention and treatment of MI.

## Materials and Methods

### Ischemia-reperfusion (I/R) injury model construction and virus injection

An I/R model was established in 8-week-old male mice (Shanghai Bikai Animal Co., Ltd., Shanghai, China) anaesthetized with tribromoethanol (1.25%, 200 μL /10 g, i.p., M2920; Nanjing Aibei Biotechnology Co., Ltd., Nanjing, China). After tracheal intubation and mechanical ventilation, a left thoracic incision was made to expose the heart. The LAD was ligated with a 7-0 suture after passing it alongside a PE-10 catheter (20-3010; Huayonbio, Shenzhen, China). Following 45 min of ischemia, reperfusion was initiated by catheter removal. The wound was sutured, and the mice were monitored postoperatively. One week prior to I/R induction, the mice received a tail vein injection of the AAV9-CDC5L overexpression vector, with or without the AAV9-FGF10 knockdown vector (1 × 10¹² v.g./mL, 200 μL per mouse). Cardiac function was subsequently assessed by echocardiography at 4 and 28 days post-I/R. Additionally, TUNEL staining and proliferation-related immunofluorescence staining were performed on heart tissues collected at 24 h and 7 days post-I/R, respectively.

### Heart sample collection and examination

Following deep anaesthesia induced by 1.2% tribromoethanol (200 μL/10 g body weight), the mice were euthanized, and the thoracic cavity was opened. The chest wall adhesions were carefully dissected with fine scissors, and the heart was excised using forceps. Immediately after extraction, the heart sample was gently rinsed in PBS to remove residual blood and prevent clotting. After thorough perfusion, the adherent tissue was carefully removed from the heart with microsurgical instruments to preserve its structural integrity.

Heart tissues designated for immunofluorescence and Masson’s trichrome staining were fixed overnight in 4% paraformaldehyde and subsequently stored in 70% ethanol. The tissues for EdU staining were preserved in 10% sucrose solution. For RNA and protein extraction, the left ventricle was dissected into three distinct regions as follows: the infarcted zone myocardium, the border zone myocardium (within 1–2 mm of the infarct border), and the remote zone myocardium (distant from the infarct area). Each tissue sample was blotted dry on filter paper, placed into cryotubes, and stored in liquid nitrogen.

### Echocardiography analysis

In 8-week-old male mice, AAV9:cTNT-CDC5L or AAV9:cTNT-CON, alone or in combination with AAV9-FGF10, was administered via tail vein injection one week prior to I/R surgery. Cardiac function was evaluated by echocardiography in each experimental group at 4 and 28 days post-I/R (dpi). Echocardiographic examinations were conducted at the Animal Experiment Center of Nanjing Medical University under inhalation anaesthesia. Cardiac parameters were acquired using a dedicated small-animal ultrasound imaging system. The measured indices included ejection fraction (EF), fractional shortening (FS), left ventricular internal dimensions at systole and diastole (LVIDs and LVIDd, respectively), interventricular septal thickness at end-systole and end-diastole (IVSs and IVSd, respectively), left ventricular posterior wall thickness at systole and diastole (LVPWs and LVPWd, respectively), and left ventricular internal diameter during early and late diastole (LVD). All measurements were performed in quadruplicate to ensure accuracy and reproducibility.

### Isolation, culture, and transduction of primary cardiomyocytes from neonatal mice

Primary cardiomyocytes were isolated from 0- to 3-day-old neonatal mice. Hearts from 100–150 pups were excised, rinsed in chilled 1× ADS solution, and minced thoroughly. The tissue fragments were digested repeatedly with prewarmed digestion solution on a horizontal shaker (180 *g* and 37°C) for 6 min each. The supernatants from each digestion were collected, and the reaction was stopped with horse serum. The combined cell suspensions were centrifuged (180 *g* and 5 min), and the pellet was resuspended in culture medium, filtered through 40-μm mesh, and subjected to differential adhesion for 45 min on coated dishes to remove fibroblasts. The nonadherent cardiomyocytes were collected and further purified by Percoll density gradient centrifugation (1800 *g*, 30 min, and 22°C). The cardiomyocyte-rich middle layer was harvested, washed, and resuspended in culture medium. After cell counting, the cardiomyocytes were plated and maintained in a CO _2_ incubator. Viral transduction or drug treatments were applied after 24–36 h of culture. For *in vitro* experiments, cultured cardiomyocytes were transduced with CDC5L-overexpressing or *CDC5L*-knockdown adenoviruses with or without *FGF10*-knockdown adenoviruses 24 h after plating. The viral titre was 1 × 10 ^10^ PFU/mL, and the viral vectors were added to the culture medium at a ratio of 2 μL/mL.

### Generation of the OGD/R model in primary cardiomyocytes

An *in vitro* model of I/R injury was established in primary neonatal mouse cardiomyocytes using OGD/R. The cells were preconditioned, cultured under standard conditions and then transferred to glucose-free and serum-free DMEM (C11995500BT; Gibco, Logan, USA). The cultures were placed in an AnaeroPACK rectangular jar with an anaerobic pouch and incubated for 8 h at 37°C to simulate ischemia. Reoxygenation was initiated by replacing the medium with glucose-containing DMEM and returning the cells to a normoxic incubator (5% CO _2_ and 37°C) for 12 h. Finally, apoptosis was assessed by TUNEL fluorescence staining.

### Immunofluorescence staining analysis

To assess proliferation and apoptosis, primary neonatal mouse cardiomyocytes on laminin-coated coverslips were fixed with 4% PFA and permeabilized with 0.5% Triton X-100. After blocking with normal goat serum (Beyotime, Shanghai, China), cells were incubated overnight with primary antibodies against cTnT (cardiomyocyte marker, ab8295; Abcam, Cambridge, UK) and either Ki-67 (9129S; CST, Boston, USA) or phospho-histone H3 (proliferation markers) (9701S; CST). For EdU detection, the Click-iT EdU (Thermo Fisher Scientific, Waltham, USA) reaction was performed prior to immunostaining. Following primary antibody incubation, a TUNEL assay (Vazyme, Nanjing, China) was conducted for apoptosis detection. Finally, cells were stained with fluorescent secondary antibodies and Hoechst before mounting.

For EdU or and TUNEL staining, mice were injected with EdU two days before sacrifice. Hearts were fixed, cryoprotected, and cryosectioned for EdU, or paraffin-embedded for TUNEL. Paraffin sections underwent deparaffinization, rehydration, and antigen retrieval. All sections were then blocked and incubated overnight with cTnT primary antibody. After washing, sections were incubated with fluorescent secondary antibodies and Hoechst. TUNEL sections were subsequently treated with Proteinase K and incubated with the TdT reaction mix. All slides were finally washed and mounted for imaging.

For both cardiomyocytes and tissue sections, images were captured using a confocal microscope. Cardiomyocytes were identified by positive cTnT staining. A cell was considered positive for proliferation (EdU, Ki67, pH3) or apoptosis (TUNEL) only if the signal co-localized with a DAPI-stained nucleus within the cTnT-positive cytoplasm. Positive cells were counted in multiple random fields and expressed as a percentage of total cardiomyocytes.

### Masson’s trichrome staining

The tissue sections were stained using a Masson’s trichrome staining kit (Solarbio, Beijing, China) following standard dehydration. In brief, rehydrated sections were stained with Weigert’s iron hematoxylin (10 min), differentiated in acid-alcohol (15 s), and rinsed in distilled water (1 min). After treatment with the bluing solution (3 min) and a second water rinse (1 min), the sections were stained with Ponceau S fuchsin solution (5 min), rinsed in weak acid working solution (1 min), and differentiated in phosphomolybdic acid (2 min), followed by another weak acid rinse (1 min). The sections were then immersed directly in aniline blue staining solution (2 min) and rinsed again in weak acid working solution (1 min). Finally, rapid dehydration was performed through three changes of 95% ethanol, three changes of absolute ethanol, and two changes of xylene (2 min each), and the samples were then mounted with resin.

### Quantitative real-time polymerase chain reaction (qRT-PCR) analysis

Myocardial tissues obtained from I/R model animals or primary cardiomyocytes from neonatal mice were homogenized in 1 mL of TRIzol reagent (G3013; Servicebio, Wuhan, China) and transferred to a 1.5 mL microcentrifuge tube. Total RNA was extracted, and the RNA concentration was measured. Total RNA (20 μg) was reverse transcribed into complementary DNA (cDNA) using the following reaction conditions: 37°C for 15 min and 85°C for 5 s. The cDNA was stored at 4°C until further use. Quantitative real-time PCR was conducted in accordance with the manufacturer’s protocol to assess the expression levels of the target genes. The PCR conditions were as follows: initial denaturation at 95°C for 5 min; 40 cycles of 95°C for 10 s and 60°C for 30 s; and a final dissociation stage. The primer sequences are listed in Table 1.

**Table:** **Table 1** Sequences of primer used in this study

Gene	Sequence (5′→3′)
*CD74*	Forward	TACTGCTGGTGTGTGTTCCC
Reverse	CGTGTCCTGGGACGATGAAA
*CPA4*	Forward	TATGTGACTGGCGCCCTTG
Reverse	ACCACTCCGCTCTATCCCTT
*EIF5*	Forward	ACCGAGAACTCTTGCAGTCG
Reverse	AGAACTGGTCTGACACGCTG
*FGF10*	Forward	CCGACACCACCAGTTCCTAC
Reverse	CTTTGACGGCAACAACTCCG
*CDC5L*	Forward	TGGCACCTGCGGTTTGATTA
Reverse	CCATGTCTATCGGGTCAGGC
*18s*	Forward	GTAACCCGTTGAACCCCATT
Reverse	CCATCCAATCGGTAGTAGCG

### Western blot analysis

Protein samples were mixed with 5× loading buffer, heated at 99°C for 10 min, and cooled on ice. The samples were stored at –20°C or used immediately. SDS-PAGE gels (stacking and separating) were prepared according to the manufacturer’s instructions (Epizyme, Shanghai, China). After the samples (30 μg) and markers (2–4 μL) were loaded into the gels, electrophoresis was performed at 80 V (stacking gel, 20–25 min) and 120 V (separating gel, ~90 min). PVDF membranes (8 cm × 5 cm; Millipore, Burlington, USA) were activated in methanol during electrophoresis. The proteins were transferred to PVDF membranes at 300 mA for 2 h or 100 V for 2 h. The membranes were blocked in 5% BSA/TBST on a shaker (80 rpm, 2–3 h, and room temperature). The membranes were then incubated overnight at 4°C with the following primary antibodies: anti-Flag (PM020; MBL, Beijing, China), anti-CDC5L (12974-1-AP; Proteintech, Wuhan, China), anti-GAPDH (60004-1-Ig; Proteintech), anti-Lamin B (12987-1-AP;Proteintech), anti-BAX (50599-2-Ig; Proteintech), anti-Bcl-2 (12789-1-AP; Proteintech), anti-FGF10 (29749-1-AP; Proteintech), anti-γ-H2AX (10856-1-AP; Proteintech), anti-YAP and anti-p-YAP (Ab76252 and ab205270; Abcam) and anti-cyclin D1 (Ab16663; Abcam) antibodies. On the following day, the membranes were washed three times with Tris-buffered saline containing Tween-20 (TBST) and incubated with diluted secondary antibodies on a shaker (60 rpm, room temperature, and 2 h). The membranes were then washed with TBST (160 rpm for 90 min; 3–4 washes) and rinsed with TBS. ECL reagent (Servicebio) was applied to the membranes, and images were acquired via a chemiluminescence system (Tanon, Shanghai, China). The band intensities were analyzed and recorded.

### Transcriptomics

Total RNA was extracted from the CON and CDC5L groups using TRIzol reagent, followed by quantification and quality assessment using a NanoDrop spectrophotometer (Thermo Fisher Scientific). For RNA sample preparation, 3 μg of total RNA was used as the starting material. RNA fragmentation was performed in Illumina proprietary fragmentation buffer containing divalent cations under elevated temperature. First-strand and second-strand cDNA were then sequentially synthesized. The remaining overhangs were converted into blunt ends through exonuclease/polymerase activities, followed by enzyme removal. After the 3′ ends of the DNA fragments were adenylated, Illumina PE adapter oligonucleotides were ligated for hybridization preparation. Library fragments were then purified using the AMPure XP system (Beckman Coulter) to select cDNA fragments 400–500 bp in length. DNA fragments with ligated adaptor molecules on both ends were enriched through 15-cycle PCR using an Illumina PCR Primer Cocktail (San Diego, USA). The PCR products were purified using the AMPure XP system (Beckman Coulter, Beverly, USA) and quantified using the Agilent high-sensitivity DNA assay on a Bioanalyzer 2100 system (Agilent, Santa Clara, USA).

The sequencing library was sequenced on the NovaSeq 6000 platform (Illumina), generating raw data in FASTQ format. The raw data were processed using Cutadapt (v1.15; https://cutadapt.readthedocs.io/en/v1.15/) software to obtain high-quality clean data for downstream analysis. Gene expression levels were quantified using HTSeq (0.9.1) by counting the mapped reads for each gene.

### Ethics approval and consent to participate

All experimental protocols were reviewed and approved by the Institutional Animal Care and Use Committee of Nanjing Medical University (ethical approval number: K-2022-052) and conformed to the Guide for the Care and Use of Laboratory Animals published by the National Institutes of Health, USA.

### Statistical analysis

The data were analyzed using SPSS 22 statistical software. Data are presented as the mean ± SEM. All *in vitro* and *in vivo* tests were conducted with three or more biological replicates. Student’s *t* test was used to compare the differences between two groups, whereas one-way ANOVA with post hoc Tukey’s multiple comparisons test was used to analyze the differences among three or more groups. For *in vivo* time course data, two-way ANOVA with post hoc Tukey’s multiple comparisons test was used. *P* < 0.05 was considered to indicate statistical significance.

## Results

### CDC5L expression is upregulated after cardiac I/R injury

**Figure 1:**
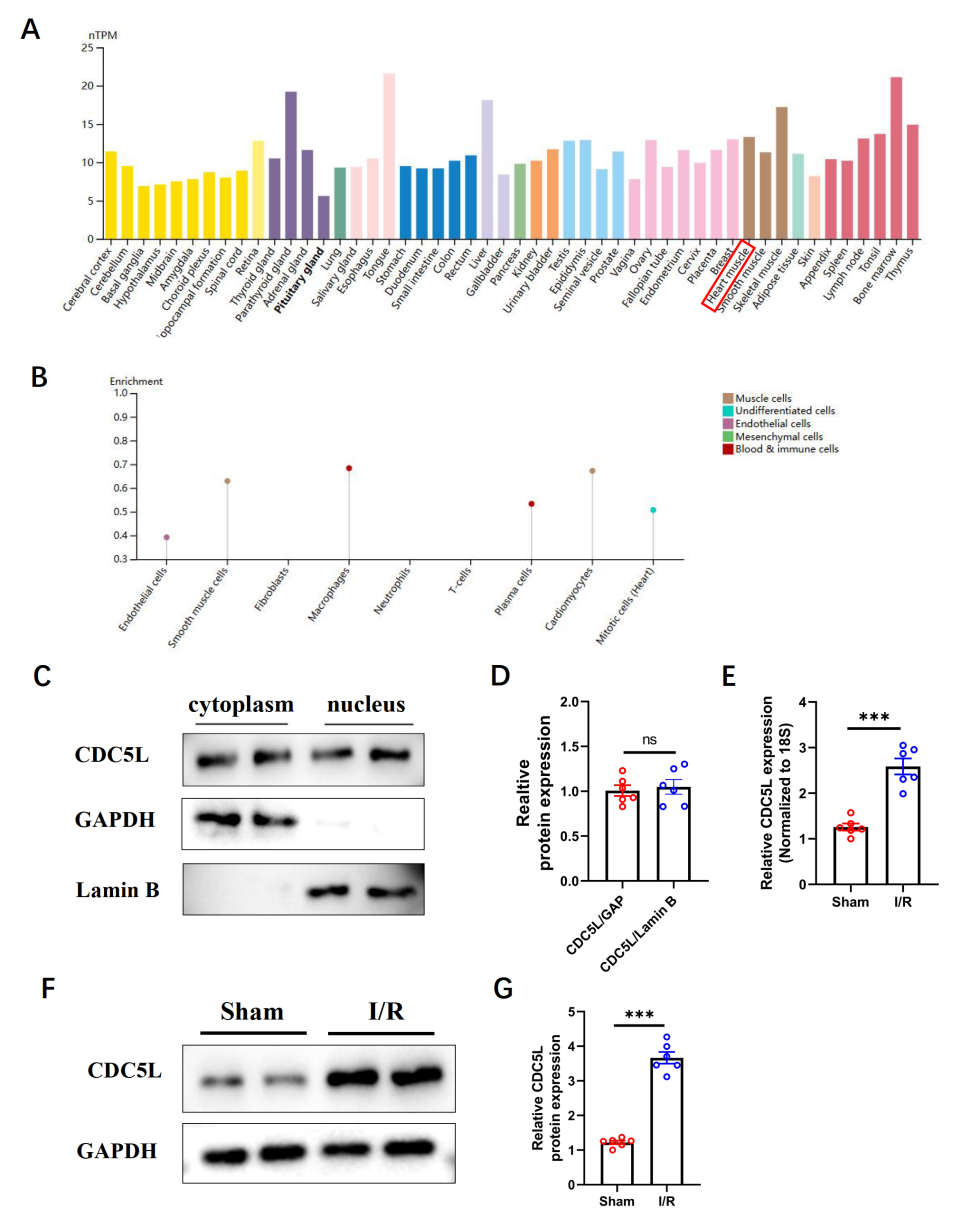
Figure 1 CDC5L expression is upregulated after cardiac I/R injury (A,B) The CDC5L expression characteristics were analyzed in human cardiac muscle and cells using the Human Protein Atlas ( https://www.proteinatlas.org/). (C,D) Western blot analysis was used to detect CDC5L expression in both the cytoplasm and nucleus of mouse cardiomyocytes. (E) qRT-PCR analysis was performed to measure the transcription levels of CDC5L in ventricular muscle after I/R injury. (F‒G) Western blot analysis was used to determine CDC5L expression in ventricular muscle after I/R injury. Each experiment was repeated three times. n = 6 in each group in the qRT-PCR experiment. ***P < 0.001.

Analysis of the Human Protein Atlas revealed that CDC5L is expressed in multiple human organs, including the heart, indicating that it is not a tissue-specific protein ( Figure 1A). CDC5L expression was predominantly localized in cardiomyocytes, with lower levels observed in cardiac fibroblasts and immune cells ( Figure 1B). Primary cardiomyocytes were isolated from neonatal mice, and cytoplasmic and nuclear proteins were extracted through subcellular fractionation. Western blot analysis confirmed that CDC5L was present in both the cytoplasm and nucleus ( Figure 1C,D). Finally, an I/R injury model was established in adult mice. Ventricular tissues were collected 48 h post-injury, and qRT-PCR and western blot analysis revealed significant upregulation of CDC5L expression at both the transcriptional and translational levels in the I/R group compared with the control group ( Figure 1E–G).

### CDC5L regulates the proliferation of mouse cardiomyocytes *in vitro*

**Figure 2:**
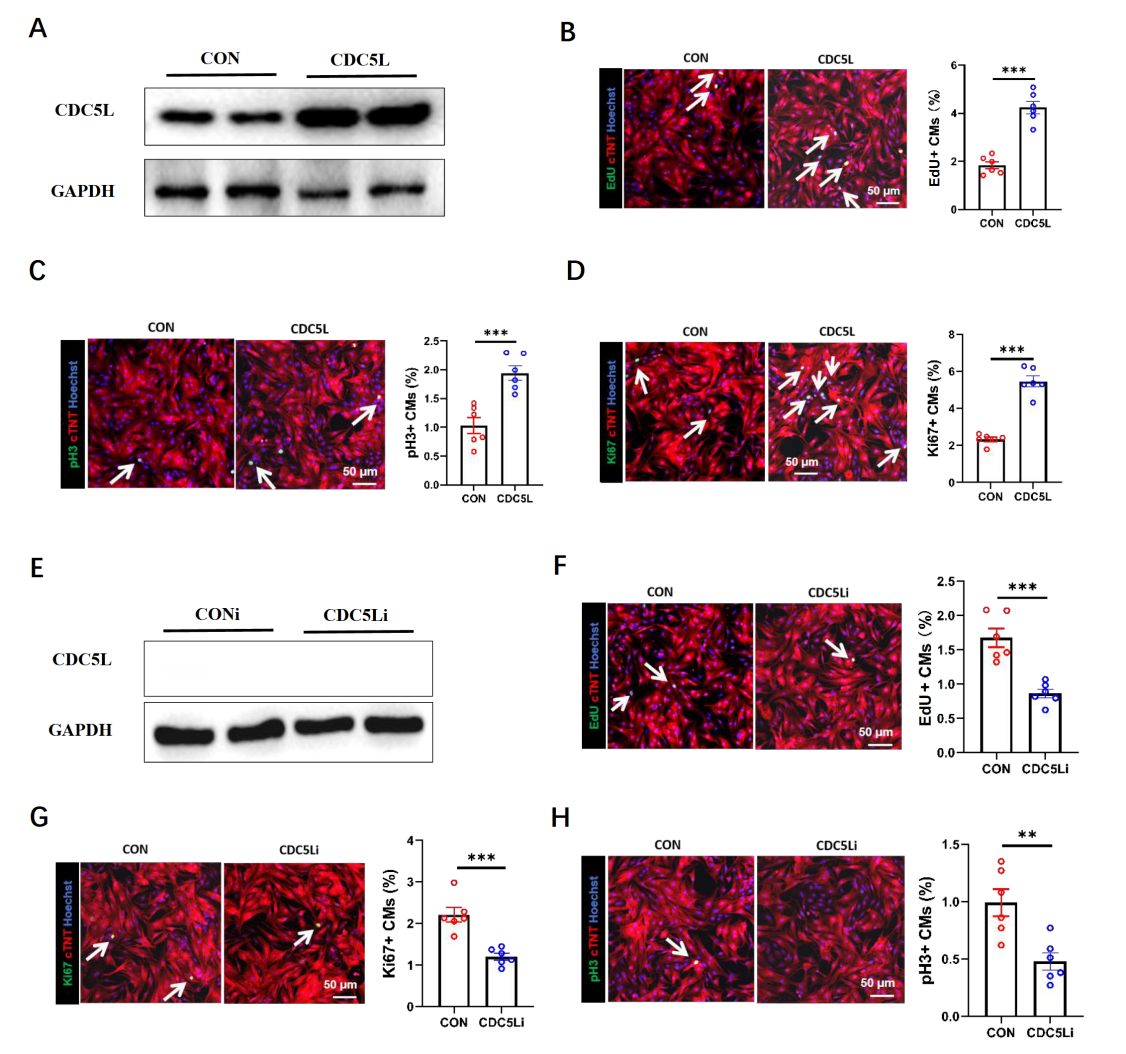
Figure 2 CDC5L promotes cardiomyocyte proliferation *in vitro* (A) Western blot analysis was used to confirm CDC5L overexpression in neonatal mouse cardiomyocytes transduced with the CDC5L adenovirus for 48 h. (B–D) Immunofluorescence staining showing increased proliferation markers, including EdU+ (DNA synthesis, B), Ki67+ (cell cycle activity, C), and pH3+ (mitotic activity, D), in CDC5L-overexpressing cardiomyocytes. (E) Western blot analysis was used to confirm the CDC5L knockdown efficiency in cardiomyocytes transduced with the CDC5L-targeting adenovirus. (F–H) Compared with those in control cardiomyocytes, the levels of proliferation markers, including EdU+ (F), Ki67+ (G), and pH3+ (H), were lower in CDC5L-knockdown cardiomyocytes. Each experiment was repeated three times. **P < 0.01 and ***P < 0.001.

CDC5L is a cell cycle regulatory protein whose expression is significantly upregulated in the myocardium after I/R injury. To investigate the role of CDC5L in promoting cardiomyocyte proliferation, a CDC5L overexpression adenovirus was constructed and transduced into neonatal mouse cardiomyocytes for 48 h. The overexpression efficiency was confirmed by western blot analysis ( Figure 2A). Immunofluorescence staining revealed that CDC5L overexpression enhanced cardiomyocyte proliferation, as evidenced by increased proportions of cells positive for DNA synthesis (EdU ^+^), cell cycle activity (Ki67 ^+^), and mitosis (pH3 ^+^) ( Figure 2B–D). Conversely, cardiomyocytes were transduced with a *CDC5L* knockdown adenovirus for 48 h, and the knockdown efficiency was verified by western blot analysis ( Figure 2E). Immunofluorescence staining revealed that *CDC5L* knockdown reduced the percentage of EdU-, Ki67-, and pH3-positive cardiomyocytes ( Figure 2F–H). These results demonstrated that CDC5L, a cell cycle-associated protein, regulates cardiomyocyte proliferation.

### CDC5L impedes OGD/R-induced cardiomyocyte apoptosis *in vitro*

**Figure 3:**
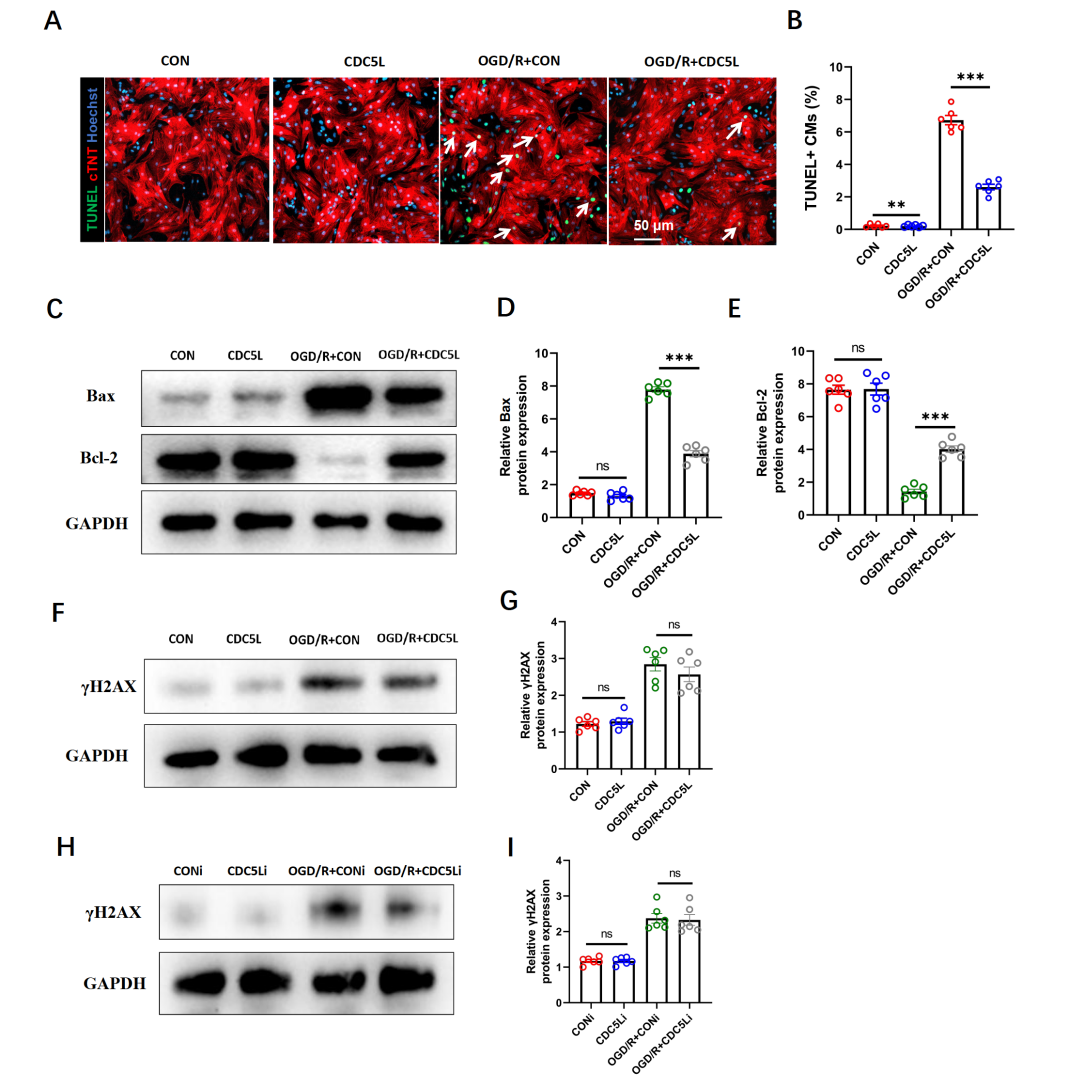
Figure 3 CDC5L attenuates OGD/R-induced cardiomyocyte apoptosis *in vitro* (A,B) TUNEL assay was used to detect the degree of apoptosis in neonatal mouse cardiomyocytes subjected to OGD/R. (C–E) Western blot analysis was used to measure the levels of the apoptosis-related proteins Bax and Bcl-2 in OGD/R-treated cardiomyocytes. (F–I) Western blot analysis was used to measure the expression of γH2AX, a DNA damage-related protein, in OGD/R-induced cardiomyocytes. Each experiment was repeated three times. ***P < 0.001.

On the basis of the important role of apoptosis in myocardial I/R injury repair and previous evidence linking CDC5L to apoptosis regulation and DNA repair, we investigated whether CDC5L modulates cardiomyocyte apoptosis and DNA repair under I/R injury. An *in vitro* OGD/R model was established in neonatal mouse cardiomyocytes using an AnaeroPACK system. The cells were transduced with a CDC5L-overexpressing adenovirus for 24 h, followed by 8 h of OGD/R in serum-free medium at 37°C and then 12 h of reoxygenation in complete medium. TUNEL staining revealed a significant increase in apoptosis after OGD/R, which was markedly attenuated by CDC5L overexpression ( Figure 3A,B). Consistently, western blot analysis of apoptosis-related proteins revealed that CDC5L overexpression significantly suppressed the protein expression levels of pro-apoptotic Bax and increased the protein expression levels of anti-apoptotic Bcl-2 in cardiomyocytes under OGD/R conditions ( Figure 3C–E). Given the reported role of CDC5L in DNA damage repair, we further evaluated the effect of CDC5L on γ-H2AX, a marker of DNA double-strand breaks. Although OGD/R induced γ-H2AX expression, neither the overexpression nor the knockdown of *CDC5L* altered the expression level of γ-H2AX ( Figure 3F–I).

### CDC5L overexpression promotes cardiac injury repair post-I/R injury *in vivo*

**Figure 4:**
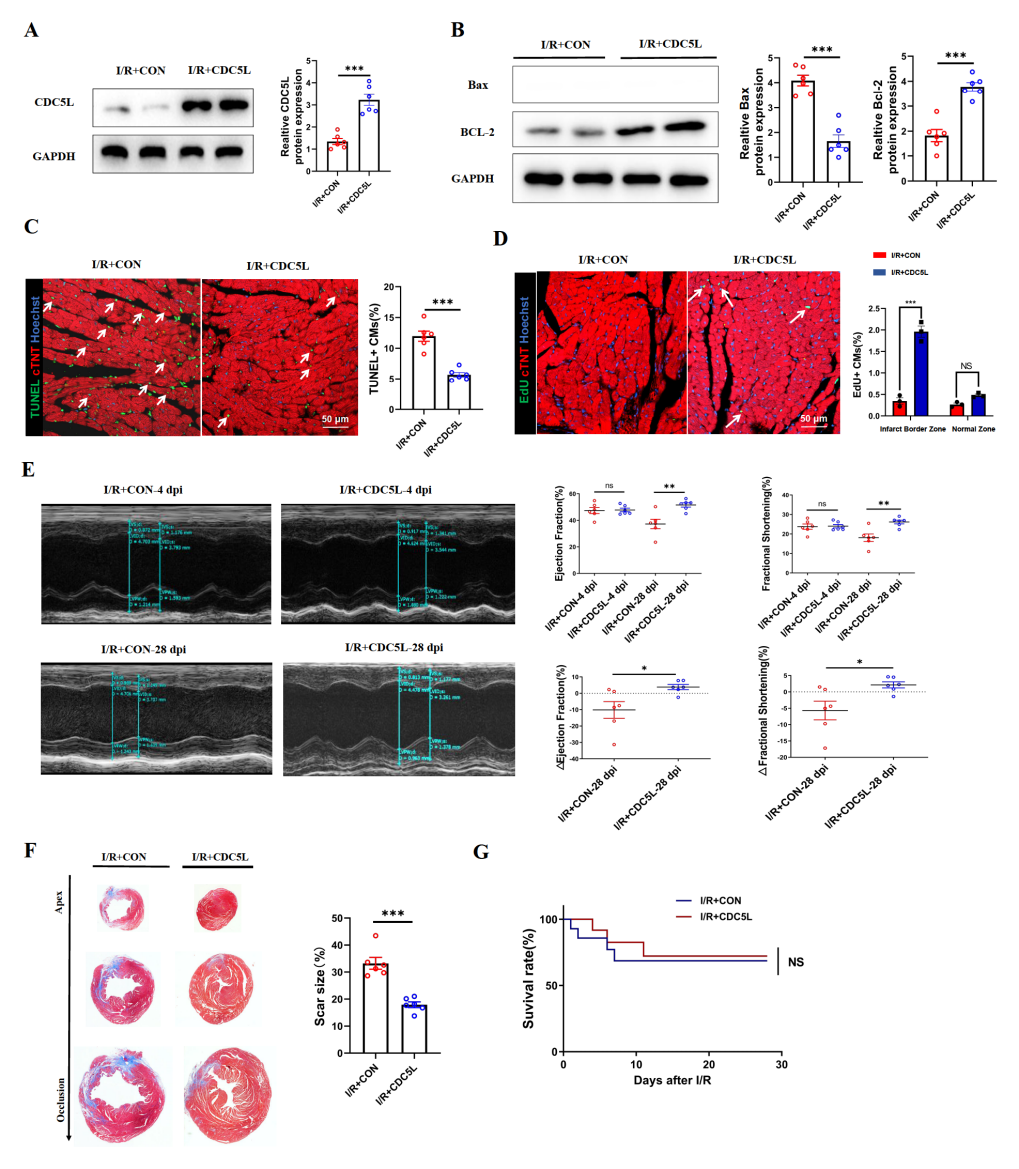
Figure 4 CDC5L promotes mouse cardiac injury repair after I/R (A) Western blot analysis was used to confirm CDC5L overexpression in mouse cardiac tissue 24 h post-I/R injury following AAV9-CDC5L tail vein injection. (B) Western blot analysis was used to confirm decreased Bax protein level and increased Bcl-2 protein level in the CDC5L group. (C) TUNEL staining of the infarct border tissue revealed reduced numbers of apoptotic cardiomyocytes in the CDC5L group. (D) EdU immunofluorescence 7 days post-I/R revealed increased proliferation of border zone cardiomyocytes in CDC5L-treated mice. (E) Echocardiography 28 days post-I/R demonstrated preserved EF and FS in the CDC5L group. (F) Masson’s trichrome staining revealed reduced fibrosis in CDC5L-treated mice compared with I/R controls. (G) Comparison of survival rates between the AAV9:CON group and the AAV9:CDC5L group after IR surgery. Each experiment was repeated three times. *P < 0.05, **P < 0.01, and ***P < 0.001.

To assess the therapeutic potential of CDC5L in myocardial repair, adult mice were administered with AAV9-CDC5L or control virus via tail vein injection 7 days before being subjected to I/R injury. Western blot analysis confirmed the overexpression of CDC5L ( Figure 4A) and demonstrated that CDC5L significantly attenuated I/R-induced cardiomyocyte apoptosis ( Figure 4B). Consistent with these findings, immunofluorescence staining of the infarct border zone 24 h post-I/R revealed a markedly lower proportion of apoptotic cardiomyocytes in the CDC5L-treated group than in the control group ( Figure 4C). At seven days post-I/R, EdU staining revealed a significantly greater percentage of EdU-positive cardiomyocytes in the infarct border tissue of the CDC5L group, suggesting enhanced cardiomyocyte proliferation ( Figure 4D). Echocardiography performed 28 days post-I/R revealed that the sharp decreases in the left ventricular ejection fraction (EF) and fractional shortening (FS) observed in I/R-injured mice were significantly ameliorated in the animals treated with AAV9-CDC5L ( Figure 4E). Additionally, Masson’s trichrome staining revealed extensive myocardial fibrosis in the I/R group, whereas mice that received AAV9-CDC5L exhibited a substantially reduced fibrotic area ( Figure 4F). Collectively, these *in vivo* findings suggest that CDC5L promotes functional recovery and attenuates fibrotic scarring following I/R injury, potentially through mediating cardiomyocyte proliferation and apoptosis.

### Transcriptome screening for downstream targets of CDC5L

**Figure 5:**
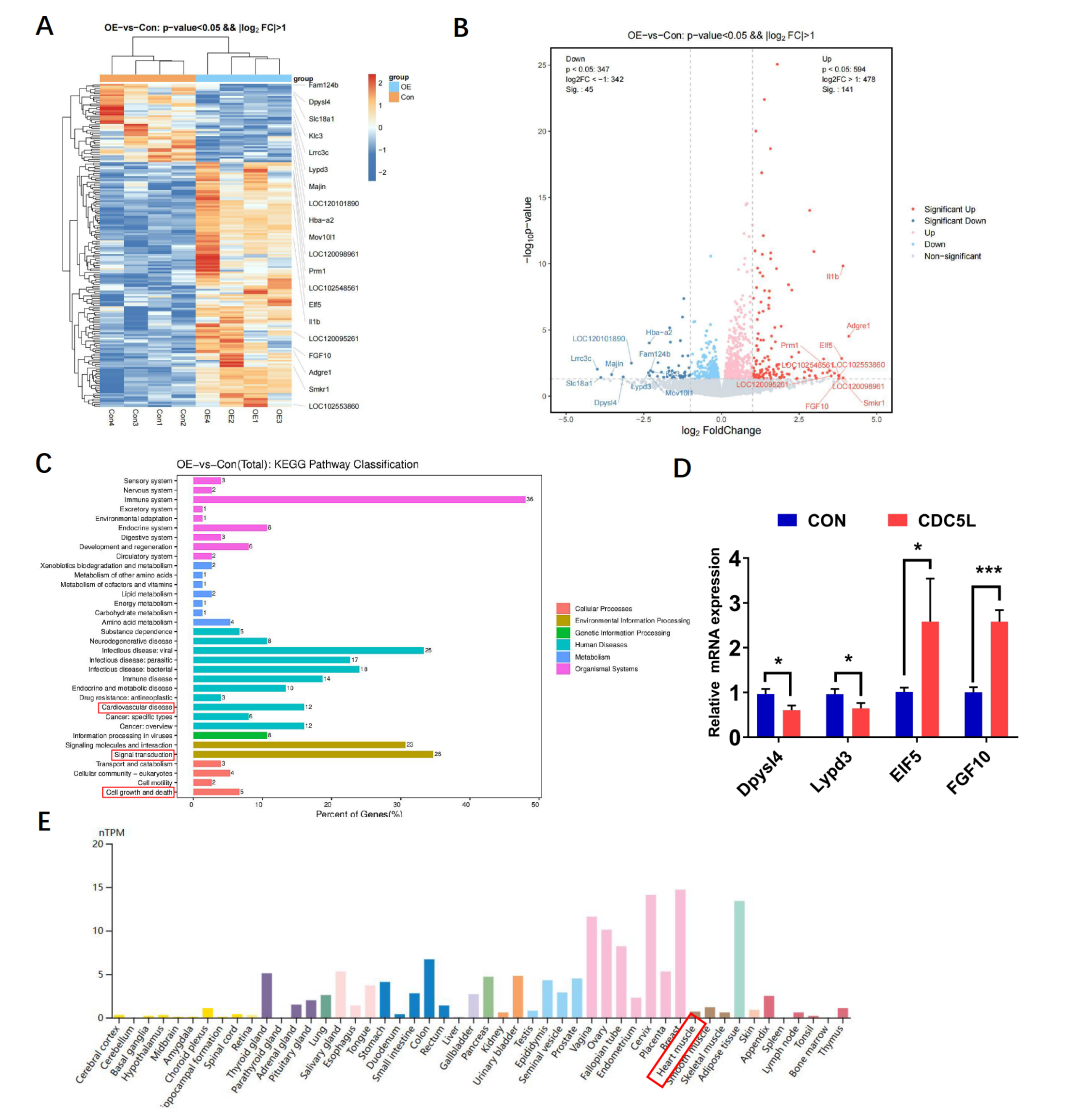
Figure 5 Transcriptome screening for downstream targets of CDC5L (A,B) Differential gene expression analysis (A) and volcano plot (B) of neonatal mouse cardiomyocytes transduced with CDC5L-overexpressing adenovirus versus control cardiomyocytes (|log2FC| >1, P < 0.05). (C) KEGG pathway enrichment analysis of differentially expressed genes. (D) RT-PCR validation showing significant upregulation of FGF10 mRNA levels in CDC5L-overexpressing cardiomyocytes compared with control cardiomyocytes. (E) Human Protein Atlas data illustrating the tissue-specific expression of FGF10. Each experiment was repeated three times. *P < 0.05 and *** P < 0.001.

To elucidate the molecular mechanisms underlying the cardioprotective effects of CDC5L against I/R injury, neonatal mouse cardiomyocytes were transduced with a CDC5L-overexpressing adenovirus for 48 h, followed by transcriptomic analysis using RNA sequencing. Differential gene expression analysis revealed 186 significantly dysregulated genes (|log2FC| > 1, *P* < 0.05) in CDC5L-overexpressing cardiomyocytes compared with controls, comprising 141 upregulated and 45 downregulated genes. ( Figure 5A,B). KEGG pathway enrichment analysis revealed that these genes were associated with cardiovascular diseases, signal transduction, and cell growth and death pathways, and these genes were closely linked to various biological processes, such as cell proliferation and apoptosis ( Figure 5C). We focused on the significantly differentially expressed genes with strong potential to regulate cardiomyocyte proliferation and apoptosis and identified *CD74*, *CPA4* , *EIF5*, and *FGF10*. Subsequent validation experiments in CDC5L-overexpressing cardiomyocytes confirmed that *FGF10* expression was significantly upregulated compared with that in the control group ( Figure 5D). Analysis of the Human Protein Atlas revealed that FGF10 was expressed in myocardial tissue, albeit at lower levels than in other organs, such as breast and adipose tissues ( Figure 5E).

### CDC5L mediates cardiomyocyte proliferation and apoptosis *in vitro* by regulating the FGF10-YAP axis

**Figure 6:**
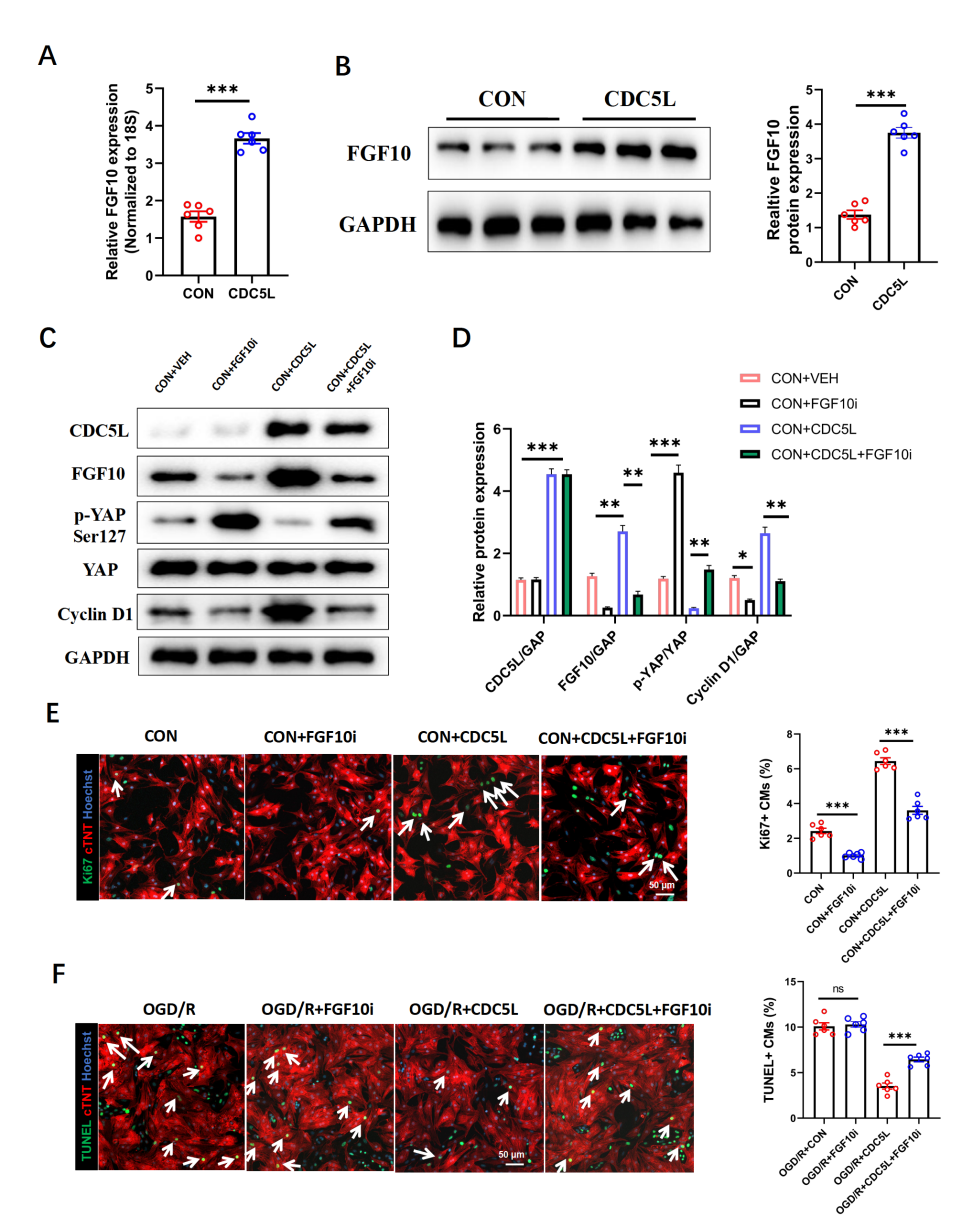
Figure 6 CDC5L plays a cardioprotective role via the FGF10-YAP axis *in vitro* (A,B) RT-PCR and western blot analysis confirmed that CDC5L overexpression increased FGF10 mRNA (A) and protein (B) expression in cardiomyocytes. (C,D) Protein expression levels of CDC5L, FGF10, p-YAP, YAP, and cyclin D1 in cardiomyocytes were detected by western blot analysis. (E) Ki67 immunofluorescence staining of OGD/R-treated cardiomyocytes. (F) TUNEL assay was used to detect the rates of apoptosis in OGD/R-treated cardiomyocytes. Each experiment was repeated three times. Data are presented as the mean ± SEM. *P < 0.05, **P < 0.01, ***P < 0.001, and ns, not significant.

There is substantial evidence that FGF10 enhances cardiac repair after myocardial infarction by inhibiting apoptosis, improving fibrosis, and promoting myocardial regeneration [16]. A previous study demonstrated that FGF10 influences myocardial regeneration via regulation of the Hippo pathway, where YAP, as its major downstream effector, plays a critical role in myocardial regeneration and repair [17]. Thus, we investigated whether CDC5L exerts its cardioprotective effects through the FGF10-YAP axis. Cardiomyocytes were transduced with a CDC5L-overexpressing adenovirus, with or without transduction of an *FGF10* knockdown adenovirus, for 48 h. Subsequent qRT-PCR and western blot analyses confirmed that FGF10 expression was upregulated upon CDC5L overexpression ( Figure 6A,B). Furthermore, CDC5L overexpression suppressed YAP phosphorylation and promoted cyclin D1 expression; however, these regulatory effects were abolished following *FGF10* knockdown ( Figure 6C,D). In OGD/R-treated cardiomyocytes, the proproliferative effect of CDC5L, as assessed by Ki67 immunofluorescence, was largely attenuated upon FGF10 inhibition ( Figure 6E). Similarly, CDC5L overexpression reduced OGD/R-induced apoptosis, but this anti-apoptotic effect was reversed by *FGF10* knockdown ( Figure 6F). Collectively, these data demonstrated that the CDC5L-mediated promotion of cardiomyocyte proliferation and suppression of apoptosis are dependent on the FGF10-YAP signaling axis.

### CDC5L promotes cardiomyocyte proliferation and improves cardiac function via modulation of the FGF10-YAP axis after I/R-induced myocardial injury

**Figure 7:**
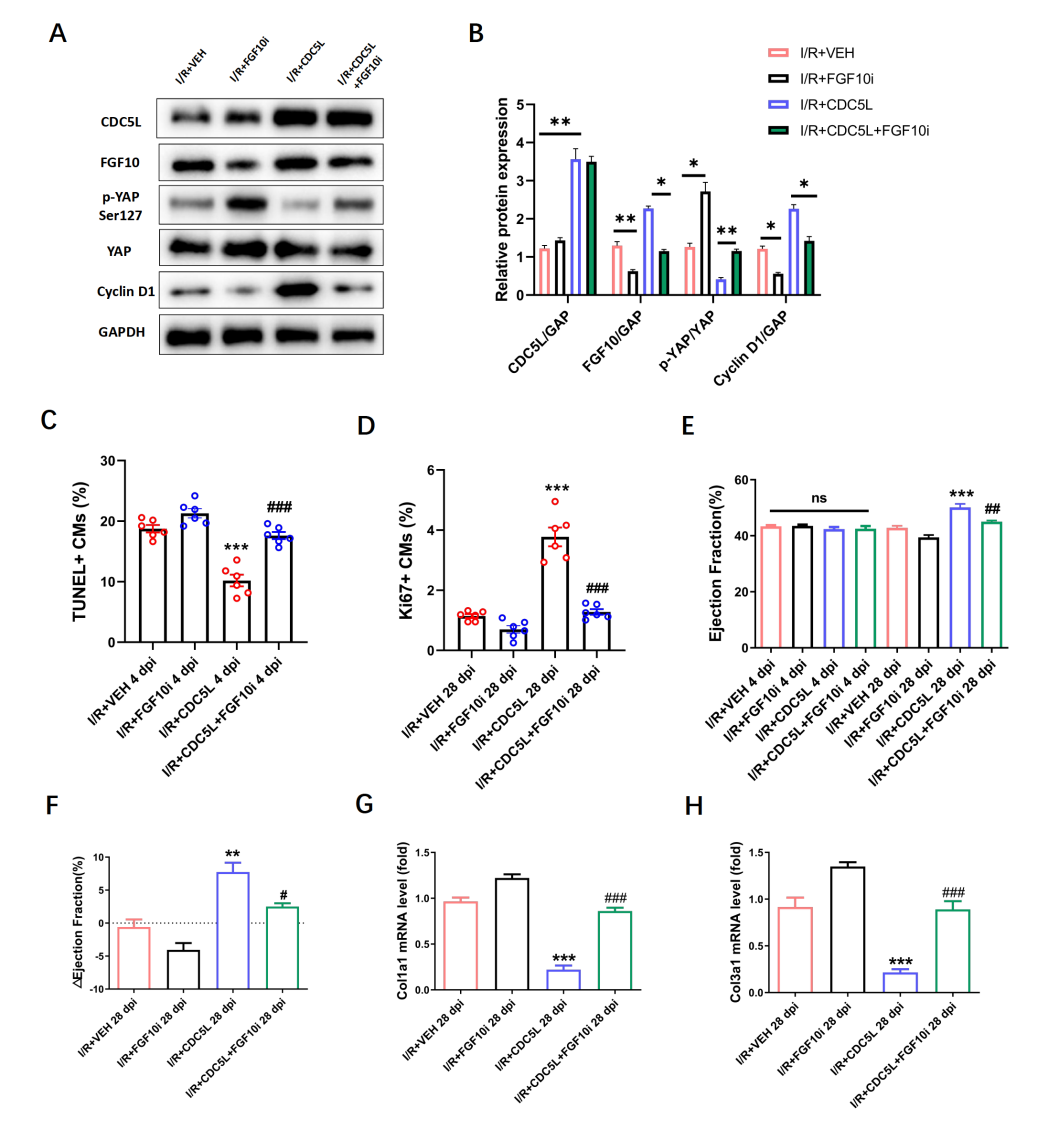
Figure 7 CDC5L protects cardiac function in an I/R mouse model via the FGF10-YAP axis (A,B) The protein expressions of CDC5L, FGF10, p-YAP, YAP, and cyclin D1 were detected by western blot analysis in an I/R mouse model. (C) TUNEL assay was used to detect the apoptosis rates in the IR + VEH, IR + FGF10i, I/R + CDC5L, and I/R + CDC5L + FGF10i groups in vivo. (D) Ki67 immunofluorescence 7 days post-I/R in the IR + VEH, IR + FGF10i, I/R + CDC5L, and I/R + CDC5L + FGF10i groups. (E,F) Echocardiography at 0 and 28 days post-I/R injury in the IR + VEH, IR + FGF10i, I/R + CDC5L, and I/R + CDC5L + FGF10i groups. (G,H) Myocardial fibrosis was determined by Col1a1 and Col3a1 expressions in the IR + VEH, IR + FGF10i, I/R + CDC5L, and I/R + CDC5L + FGF10i groups. Each experiment was repeated three times. Data are presented as the mean ± SEM. *P < 0.05, **P < 0.01, and ***P < 0.001 vs the IR + VEH group at 28 dpi. #P < 0.05 and ###P < 0.001 vs the IR + CDC5L group at 28 dpi. ns, not significant.

To further validate the role of the CDC5L-FGF10-YAP axis in regulating cardiomyocyte proliferation and cardiac repair after I/R injury, we overexpressed CDC5L using AAV9 with or without *FGF10* knockdown. Western blot analysis of cardiac tissues harvested 24 h post-I/R revealed that CDC5L overexpression upregulated FGF10 and cyclin D1 expressions but suppressed YAP phosphorylation; these changes in expression were abolished by *FGF10* knockdown ( Figure 7A,B). At 24 h post-I/R, TUNEL assay revealed that CDC5L significantly attenuated cardiomyocyte apoptosis, whereas this protective effect was reversed in the CDC5L + FGF10i group ( Figure 7C). In addition, Ki67 immunofluorescence staining demonstrated that the pro-proliferative effect of CDC5L on border-zone cardiomyocytes was abolished upon *FGF10* knockdown ( Figure 7D). Furthermore, echocardiography performed 28 days after I/R injury revealed that the marked reductions in the ejection fraction (EF) and fractional shortening (FS) observed in I/R-injured mice were ameliorated in the I/R + AAV9-CDC5L group; however, this protective effect was blocked when FGF10 was knocked down ( Figure 7E,F). Finally, RT-PCR analysis revealed that the transcript levels of *col1a1* and *col3a1*, which are markers of myocardial fibrosis, were significantly decreased in the CDC5L-overexpressing group; however, this anti-fibrotic effect of CDC5L was largely reversed following *FGF10* knockdown ( Figure 7G,H).

**Figure 8:**
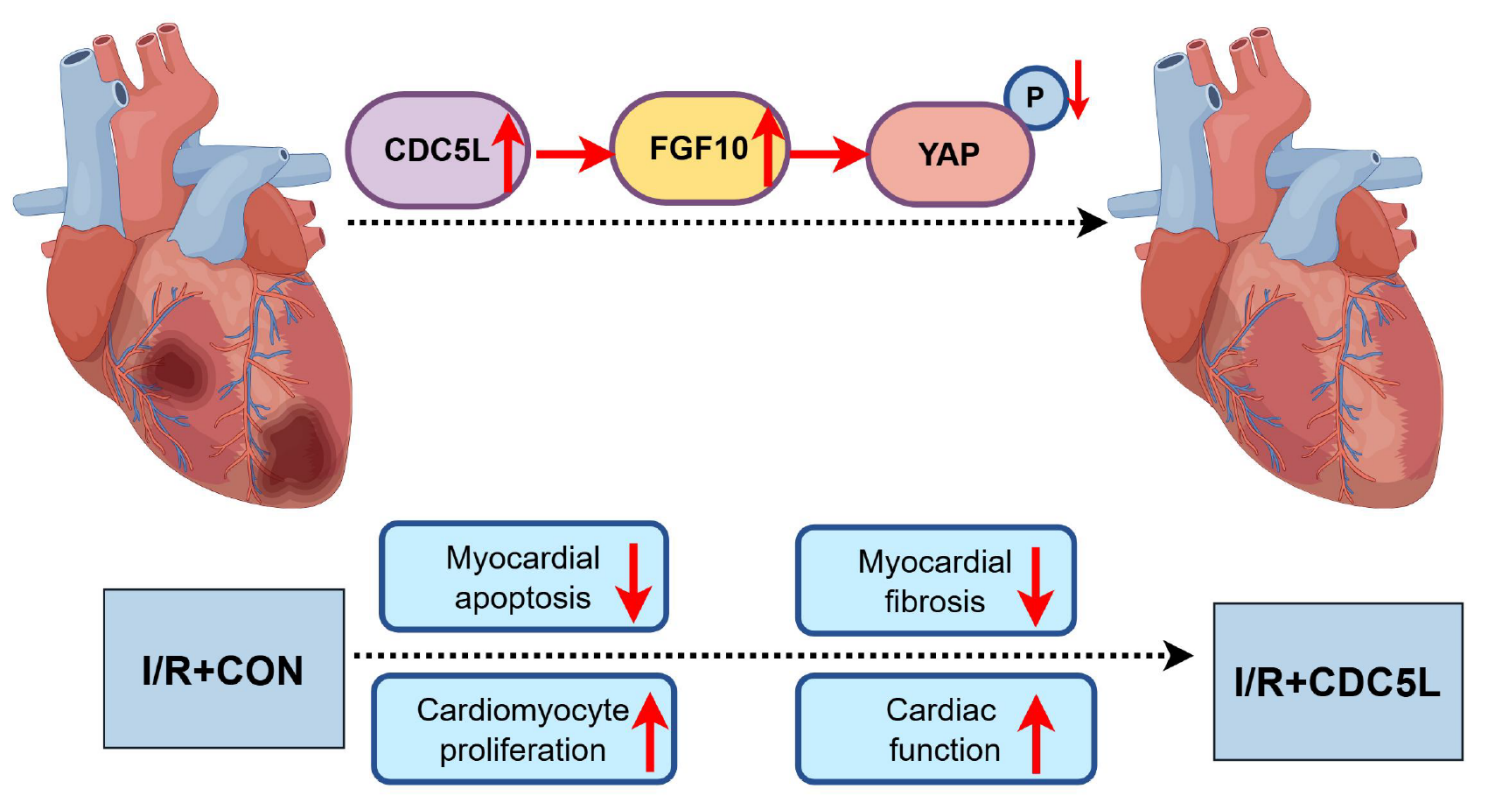
Figure 8 Schematic diagram of CDC5L-related cardioprotection and the underlying molecular mechanism in mouse models of I/R injury CDC5L-mediated FGF10 transactivation promotes cardiomyocyte proliferation and ameliorates I/R injury by driving YAP-dependent gene expression (e.g., cyclin D1).

On the basis of the above findings, a schematic model summarizing the FGF10-dependent cardioprotective role of CDC5L and its underlying molecular mechanism in mouse models of I/R injury is proposed ( Figure 8).

## Discussion

In the present study, we revealed that CDC5L, which acts as a regulatory gene of *FGF10*, facilitates cardiomyocyte proliferation both in neonatal mice and in the MI border zone of adult mice. Additionally, upregulation of CDC5L markedly ameliorated OGD/R-induced cardiomyocyte apoptosis *in vitro* and in the peri-infarct zone in adult mice, thus contributing to improvements in myocardial repair and cardiac function after ischemia‒reperfusion injury. CDC5L promotes a reduction in acute-phase infarct size and long-term fibrous scar size after I/R injury, indicating that CDC5L alleviates cardiac remodelling after MI. Mechanistically, the beneficial effects of CDC5L are mediated through the FGF10 pathway, providing a new perspective for the prevention and treatment of MI.

Although ischemia-reperfusion and other treatments used in clinical practice help improve symptoms and restore blood supply, they cannot fundamentally renew or repair the necrotic myocardium [18]. The adult mammalian heart is a terminally differentiated organ and does not have regenerative ability [19]. Research on myocardial regeneration has focused on understanding how to promote the endogenous regeneration of adult cardiomyocytes and elucidating the underlying mechanisms involved. CDC5L is an important transcription factor that regulates human gene expression, and CDC5L independently and mutually regulates the expression of certain core genes [20]. CDC5L, a key subunit of the PRP19 complex, is involved in RNA spliceosome activation and pre-mRNA splicing processes and plays vital roles in the cell cycle, apoptosis and DNA damage repair [ 13 , 14]. In the present study, compared with that in the control group, the expression of CDC5L in the ventricular muscle tissue of mice after I/R was significantly upregulated. These findings suggest that this upregulation represents an intrinsic self-protective response to injury and is insufficient to exert cardioprotective effects, a phenomenon that is consistent with the findings of other studies [ 21, 22]. We hypothesized that substantial expression of CDC5L in the myocardium significantly promotes myocardial regeneration and repair. Consistent with our hypothesis, *in vitro* functional gain and loss experiments revealed that CDC5L overexpression markedly accelerated cardiomyocyte proliferation but *CDC5L* knockdown decreased cardiomyocyte proliferation, as evidenced by the proportion of cardiomyocytes reentering the cell cycle (Ki67 ^+^), DNA synthesis (EdU ^+^), and mitosis-positive CMs (pH3 ^+^). These data suggest that CDC5L plays a regulatory role in cell proliferation. In contrast, the present study revealed that CDC5L overexpression did not affect the expression of γ-H2AX, a DNA damage marker protein, in OGD/R-induced cardiomyocytes. These results suggest that the protective effects of CDC5L are not mediated through DNA damage repair. In addition, the present study demonstrated that CDC5L inhibited cardiomyocyte apoptosis after OGD/R and I/R injury both *in vitro* and *in vivo*. These results suggest that CDC5L promotes myocardial injury repair by simultaneously promoting myocardial cell proliferation and inhibiting apoptosis.

By exploring the underlying mechanism, we identified FGF10 as a potential downstream target of CDC5L through transcriptome bioinformatics analysis. Through *in vitro* and *in vivo* experiments, we further confirmed that CDC5L significantly upregulated the expression of FGF10 and cyclin D1 but decreased the level of phosphorylated YAP. As a member of the FGF family, FGF10 is a typical paracrine fibroblast growth factor that is vital for various physiological processes, such as embryonic development, organ formation, and tissue repair [23]. The FGF10 protein typically activates intracellular signalling pathways by binding to specific cell surface receptors, thereby regulating cell proliferation, differentiation, and migration [ 23, 24]. Previous studies have shown that FGF10 regulates the YAP pathway, which is involved in cardiomyocyte apoptosis and myocardial regeneration and repair after myocardial infarction [ 17, 25]. The present study revealed that YAP is a downstream regulatory target of FGF10. Research on cardiovascular disease has shown that FGF10 is essential for the development of the myocardium, and the absence or expression of FGF10 can lead to the occurrence of congenital heart disease [26]. According to previous reports, FGF10 overexpression in adult mouse myocardial tissue promotes the entry of myocardial cells into the cell cycle, suggesting that FGF10 may be a potential target involved in myocardial repair. Using a mouse model of myocardial infarction, researchers have directly injected exogenous FGF10 into the ischemic zone to increase its level, thereby reducing myocardial damage and restoring heart function [ 16, 17]. Therefore, maintaining or even increasing the expression level of FGF10 may help myocardial tissue resist acute injury. The present study confirmed that CDC5L promotes cardiomyocyte proliferation and inhibits apoptosis in the peri-infarct zone after myocardial infarction. We further explored the role of the FGF10-YAP pathway as a potential downstream target of CDC5L through rescue experiments, which demonstrated that CDC5L exerts cardioprotective effects by regulating FGF10 expression.

In summary, the use of CDC5L as an intervention strategy simultaneously mitigated acute apoptotic injury and long-term fibrotic scarring, significantly improving cardiac remodelling and functional recovery. The present findings highlight the novel therapeutic potential of CDC5L in myocardial injury repair via regulation of the FGF10 signaling pathway, and we establish for the first time the molecular bridging role of the CDC5L-FGF10-YAP regulatory axis in myocardial regeneration. The present study provides a theoretical foundation and suggests potential therapeutic strategies for overcoming the dilemma of adult cardiomyocyte terminal differentiation, developing endogenous regenerative mechanisms, and alleviating cardiomyocyte apoptosis after myocardial infarction.

## Supporting information

25751Table_1(1)
