# Supplementary material for: CDC5L facilitates cardiomyocyte proliferation and ameliorates myocardial ischemia-reperfusion injury via modulation of the FGF10-YAP axis: CDC5L facilitates cardiomyocyte proliferation and ameliorates myocardial I/R injury
Source: Acta Biochim Biophys Sin (Shanghai). 2025 Nov 14;57(12):2110–23. doi: 10.3724/abbs.2025213 (PMC12747968; doi:10.3724/abbs.2025213)
Supplement: 25751Table_1(1) [file 25751Table_1(1).docx]

**Table 1. Sequences of primer used in this study**

| Gene | Sequence (5′→3′) | |
| --- | --- | --- |
| *CD74* | Forward | TACTGCTGGTGTGTGTTCCC |
|  | Reverse | CGTGTCCTGGGACGATGAAA |
| *CPA4* | Forward | TATGTGACTGGCGCCCTTG |
|  | Reverse | ACCACTCCGCTCTATCCCTT |
| *EIF5* | Forward | ACCGAGAACTCTTGCAGTCG |
|  | Reverse | AGAACTGGTCTGACACGCTG |
| *FGF10* | Forward | CCGACACCACCAGTTCCTAC |
|  | Reverse | CTTTGACGGCAACAACTCCG |
| *CDC5L* | Forward | TGGCACCTGCGGTTTGATTA |
|  | Reverse | CCATGTCTATCGGGTCAGGC |
| *18s* | Forward | GTAACCCGTTGAACCCCATT |
|  | Reverse | CCATCCAATCGGTAGTAGCG |
